# Treatment during cerebral vasospasm phase—complication association and outcome in aneurysmal subarachnoid haemorrhage

**DOI:** 10.1007/s00415-022-11212-w

**Published:** 2022-06-22

**Authors:** Isabel C. Hostettler, Kornelia Kreiser, Nicole Lange, Nina Schwendinger, Dominik Trost, Samira Frangoulis, Theresa Hirle, Jens Gempt, Maria Wostrack, Bernhard Meyer

**Affiliations:** 1 Department of Neurosurgery, Klinikum Rechts Der Isar, Technical University Munich, Ismaningerstrasse 22, 81675 Munich, Germany; 2 Department of Neurosurgery, Cantonal Hospital St. Gallen, St. Gallen, Switzerland; 3 Department of Neuroradiology, Klinikum Rechts Der Isar, Technical University Munich, Munich, Germany

**Keywords:** Aneurysmal subarachnoid haemorrhage, Cerebral vasospasm, Complications, Functional outcome, Risk factors

## Abstract

**Background:**

Aneurysm treatment during cerebral vasospasm (CVS) phase is frequently considered as particularly dangerous, mainly because of the risk of cerebral infarct.

**Objective:**

We aimed to evaluate the risk of aneurysmal subarachnoid haemorrhage (aSAH)-specific complications and functional outcome in patients treated during CVS phase.

**Methods:**

We retrospectively analysed a large, retro- and prospectively collected database of aSAH patients admitted to our department between March 2006 and March 2020. We conducted a uni- and multivariable logistic regression analysis to evaluate influencing factors on rebleeding, cerebral infarct, Glasgow Outcome Score (GOS) at discharge and mortality and assessed the rate of angiographic vasospasm on admission.

**Results:**

We included 853 patients. The majority of patients were female (66.6%), mean age was 57.3 years. Out of 853 included patients, 92 (10.8%) were treated during CVS phase, 312 (36.6%) underwent clipping and 541 (63.4%) endovascular treatment. Treatment during CVS phase was significantly associated with cerebral infarct in the multivariable logistic regression analysis, unrelated to the nature of intervention (OR 2.42, 1.29–4.54 95% CI *p*-value = 0.006). However, patients treated during CVS phase did not have increased risk of unfavourable outcome by GOS on discharge. In addition, they did not have a higher rate of rebleeding or mortality.

**Conclusions:**

Treatment during CVS phase was significantly associated with a higher rate of cerebral infarct as confirmed by imaging. This did not reflect on GOS on discharge, rebleeding, or mortality. Aneurysm treatment during CVS phase is relatively safe and should not be postponed due to the risk of rebleeding and subsequent devastating deterioration.

**Supplementary Information:**

The online version contains supplementary material available at 10.1007/s00415-022-11212-w.

## Introduction

Aneurysmal subarachnoid haemorrhage (aSAH) is a relatively rare form of stroke with a high mortality and morbidity [1–5]. Functional outcome is generally poor and influenced by several factors. The acute haemorrhage is the first factor causing brain injury, among other things by early depolarization, but complications following the acute haemorrhage further influence functional outcome after aSAH [6–9]. Complications include rebleeding, cerebral vasospasm (CVS), and cerebral infarction all of which influence functional outcome [8, 10–12].

There has been a change in treatment paradigm of ruptured intracranial aneurysm over the last decade with currently favoring early or even ultra-early occlusion [13–17]. However, sometimes early treatment might not be possible due to several reasons but mainly due to delayed presentation to hospital, most frequently the case in low grade aSAH [18–20]. Treatment during CVS phase has been subject to controversy; however, only few data exist [21–26]. Delaying aneurysm occlusion has been recommended before, especially if treatment decision has been made in favor of microsurgery, due to a presumably increased risk of cerebral infarct [27]. On the other hand, aneurysms which have been occluded allow for a more aggressive management of CVS including drug induced hypertension as the main pillar of vasospasm therapy, justifying aneurysm occlusion even during the CVS phase [28]. We hypothesize that treatment during CVS phase does not increase either likelihood for complications or unfavorable functional outcome.

We aim to evaluate the influence of treatment during CVS phase on rebleeding, cerebral infarct, in-hospital mortality and Glasgow Outcome Score (GOS) at discharge. In a second step we will evaluate the likelihood of the above-mentioned outcome factors analysed by Hunt and Hess (HH) grade to assess differences according to aSAH severity.

## Materials and methods

### Population

Patients with aSAH were prospectively and retrospectively recruited into our hospital-based registry between March 2006 and March 2020. We did not consider patients with non-aneurysmal SAH or patients with aSAH with other underlying diseases (such as flow-associated aneurysms due to an underlying arteriovenous malformation or mycotic aneurysms). We pre-specified a complete-case analyses only including patients with all outcome variables of interest available (cerebral vasospasm, rebleeding, cerebral infarct, functional outcome at discharge as well as mortality). Patients who had already been treated in an external hospital and were referred for further treatment or follow-up were not included in our analysis. We used the GOS at discharge to evaluate functional outcome [29]. For analysis purposes, we dichotomized the GOS into favourable (4–5) outcome and unfavourable (1–3) with favourable outcome as the reference group.

Our dependent variables of interest were rebleeding, cerebral infarct, in-hospital mortality and GOS at discharge. The main independent variable of interest was treatment during CVS phase which was defined as treatment during days 4 and 14 [30–32]. We defined CVS as radiologically confirmed intracranial arterial narrowing (vasoconstriction) on digital subtraction angiography, CT angiography or increase in flow velocity in the transcranial cerebral doppler (TCD) for patients who did not undergo CTA, MRA or DSA [33–35].

### Radiological data

We defined rebleeding as a repeated haemorrhage occurring from the ruptured aneurysms before its occlusion, confirmed by imaging. We defined cerebral infarct as radiologically proven newly developed infarct during hospitalization [36]. We assessed the Barrow Neurological Institute (BNI) scale as it has been proven to be superior to the frequently used Fisher score [37]. Available neuroimaging was evaluated by our neuroradiological department.

### Statistical analysis

Categorical variables are presented as count and percentage. Continuous variables are presented as mean with standard deviation (SD) for normally distributed data or, if conditions for normal distribution were not met, as median with interquartile range (IQR). We conducted a univariable analysis based on which the multivariable analysis was performed and adjusted the multivariable logistic regression model with the prespecified variables age and treatment during CVS phase as well as variables with a *p*-value of <  = 0.1. We first assessed influence of treatment during CVS phase in the overall cohort followed by a subgroup analysis based on HH grade to evaluate differences based on aSAH severity. We combined patients with HH grade 1 and 2 and set the level of statistical significance to 5% (*p*-value = 0.05). We investigated whether there was a significant interaction between intracerebral haemorrhage (ICH) and intraventricular haemorrhage (IVH). In none of the analysis did the interaction between these two variables reach the pre-specified threshold for interactions of *p* < 0.001 and was, therefore, not included in the models[38].

Statistical analysis was performed using STATA 15 (StataCorp. 2011. Stata Statistical Software: Release 15. College Station, TX: StataCorp LP). We report this study following the STROBE (Strengthening the Reporting of Observational Studies in Epidemiology) guidelines.

### Ethical approval

This study was approved by the local Research Ethics committee (186/20S). As no patient identifiable data is presented, no specific patient consent was needed. The study was performed in accordance with the ethical standards as laid down in the 1964 Declaration of Helsinki and its later amendments.

## Results

We included a total of 853 patients. The majority of our patients were female (586, 66.6%), 285 (33.4%) were male. Mean age in our cohort was 57.3 years (14.4 SD). Occlusion of the ruptured aneurysm was done during the CVS phase in 92 patients (10.8%), with 50 of them (54.4%) suffering from CVS during the time they were treated. The majority of our patients was treated by coiling (539, 63.2%). See Table 1 for baseline characteristics.

**Table 1:** Patients in CVS phase (d4-14)

	All, *N* = 853	Treated during CVS phase, *N* = 92	Treated outside CVS phase, *N* = 761
Age, mean (SD)	57.3 (14.3)	56.3 (14)	57.4 (14.3)
Female sex, *N* (%)	568 (66.6)	52 (56.5)	516 (67.8)
Treatment during CVS phase	92 (10.8)		
HH, *N* (%)			
1	116 (13.6)	33 (35.9)	83 (10.9)
2	286 (33.5)	42 (45.7)	244 (32.1)
3	185 (21.7)	11 (12)	174 (22.9)
4	152 (17.8)	6 (6.5)	146 (19.2)
5	114 (13.4)	0	114 (15)
Clipping, *N* (%)	314 (36.8)	32 (34.8)	280 (36.8)
Coiling, *N* (%)	539 (63.2)	60 (65.2)	481 (63.2)
Complications, *N* (%)			
Rebleeding	15 (1.8)	1 (1.1)	14 (1.8)
CVS	489 (57.3)	50 (54.4)	439 (57.7)
Spasmolysis, angioplasty	94 (11)	11 (12)	83 (10.9)
Infarction	163 (19.1)	18 (19.6)	145 (19.1)
Decompressive craniectomy	122 (14.3)	3 (3.3)	119 (15.6)
Ventriculo-peritoneal shunt insertion	211 (24.7)	12 (13)	199 (26.2)
Mortality	118 (13.8)	5 (5.4)	113 (14.9)
GOS at discharge, *N* (%)			
1	118 (13.8)	5 (5.4)	113 (14.9)
2	103 (12.1)	6 (6.5)	97 (12.8)
3	137 (16.1)	7 (7.6)	130 (17.1)
4	149 (16.4)	11 (12)	129 (17)
5	355 (41.6)	63 (68.5)	292 (38.4)

*CVS* cerebral vasospasm, *GOS* Glasgow Outcome Score, *HH* Hunt and Hess grade, *SD* standard deviation

We did not find a significant difference between patients treated during CVS phase with regards to treatment modality.

### Rebleeding

BNI score (overall *p*-value 0.006) and ICH (OR 2.76, 0.99–7.7 95% CI *p* = 0.05) measured on initial CT scan were significantly associated with aneurysm rebleeding in the univariable analysis. Based on this univariable analysis the multivariable analysis was adjusted with the pre-specified variables age and treatment during CVS phase, in addition to BNI score and ICH. Treatment during CVS phase was not significantly associated with rebleeding (OR 0.89, 0.11–7.1 95% CI *p*-value = 0.91; Table 2). Both BNI score and ICH remained significantly associated with rebleeding in the multivariable analysis (overall *p*-value 0.009; OR 3.08, 1.07–8.85 95% CI *p*-value = 0.04, respectively).

**Table 2:** Multivariable analysis rebleeding

	OR	95% CI	*P*-value
Treatment during CVS phase	0.89	0.11–7.1	0.91
Age	1.01	0.98–1.05	0.46
BNI score			**0.009**
1	None		
2	0.22	0.05–0.92	
3	0.11	0.03–0.46	
4	0.05	0.01–0.51	
5 (reference group)			
ICH	3.08	1.07–8.85	**0.004**

Significant *P*-values were marked in bold

*BNI* barrow neurological institute, *ICH* intracerebral haemorrhage

### Cerebral infarct

We adjusted the multivariable analysis with the pre-specified variables age and treatment during CVS as well as with BNI score, HH grade, ICH, IVH and CVS (Table 3). Treatment during CVS phase was significantly associated with cerebral infarct (OR 2.35, 1.24–4.44 95% CI *p*-value = 0.009). HH grade and CVS were also significantly associated with cerebral infarct in the multivariable analysis (overall *p*-value < 0.001 and < 0.001, respectively).

**Table 3:** Multivariable analysis cerebral infarct

	OR	95% CI	*P*-value
Treatment during CVS phase	2.35	1.24–4.44	**0.009**
Age	0.99	0.98–1.01	0.57
BNI score			0.08
1 (reference group)			
2	1.08	0.40–2.91	
3	1.75	0.67–4.56	
4	1.03	0.37–2.90	
5	2.28	0.70–7.45	
HH			** < 0.001**
1 (reference group)			
2	1.84	0.79–4.25	
3	2.98	1.24–7.18	
4	4.77	1.96–11.64	
5	7.75	3.12–19.26	
ICH	1.39	0.92–2.10	0.11
IVH	0.97	0.65–1.44	0.87
CVS	2.44	1.61–3.70	< 0.001

Significant *P*-values were marked in bold

*BNI* barrow neurological institute, *HH* Hunt and Hess grade, *ICH* intracerebral haemorrhage, *IVH* intraventricular haemorrhage

**Fig. 1:**
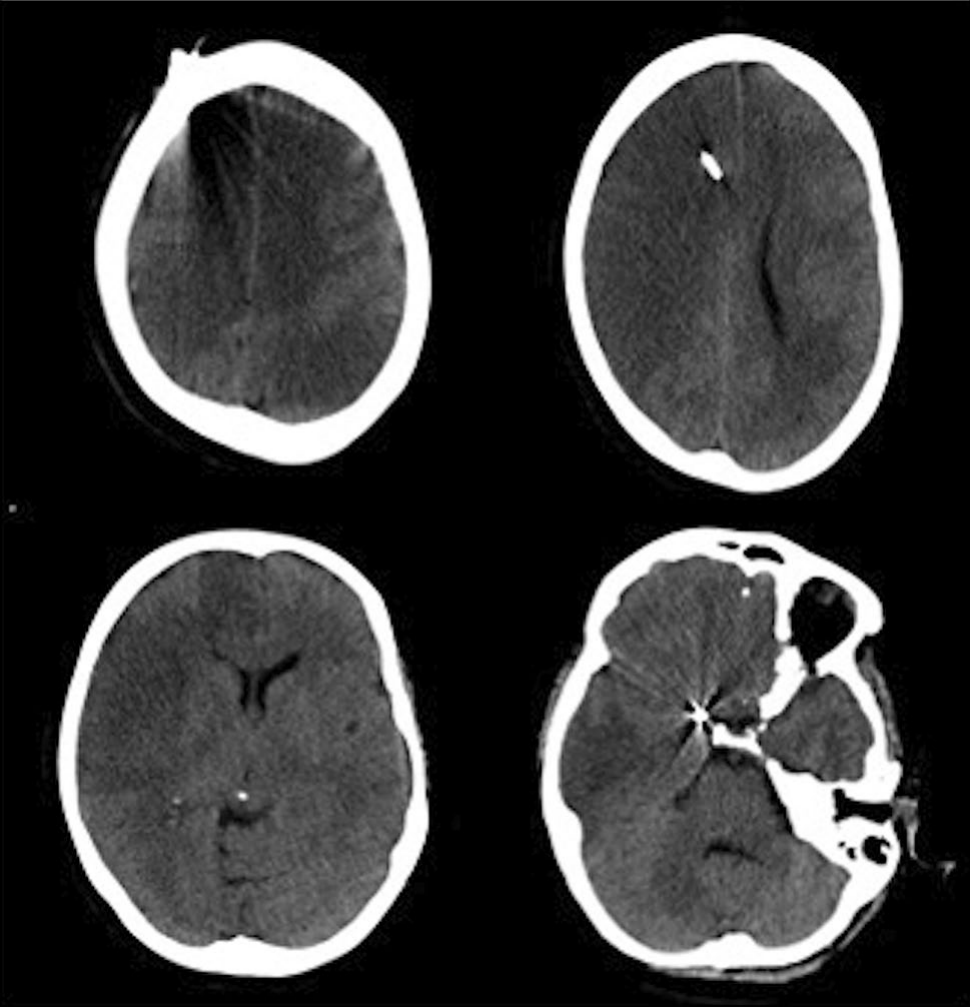
CT scan of a patient with low grade aSAH (in this case HH 1) and cerebral infarct unrelated to treatment

In the subgroup analysis by HH grade, treatment during CVS phase was still associated with cerebral infarct for lower HH grades in the multivariable analysis (HH1 and 2 OR 2.22, 0.98–5.06 95% CI *p*-value = 0.06; HH 3 OR 8.36, 2–34.88 95% CI *p*-value = 0.004) but not for HH grade 4. See Fig. 1 for an example of a patient with cerebral infarct in HH grade 1.

### Mortality

In the univariable analysis, treatment during CVS phase was inversely associated with in-hospital mortality (OR 0.33, 0.13–0.83 95% CI *p*-value = 0.02). Multivariable analysis was adjusted with the variables age, treatment during CVS phase, BNI score, HH, ICH and IVH. Age (OR 1.04, 1.02–1.05 95% CI *p*-value < 0.001) and HH grade (overall *p*-value < 0.001; Table 4) were significantly associated with mortality in the multivariable analysis. Treatment during CVS phase, however, did not remain significantly associated with in-hospital mortality in the multivariable analysis.

**Table 4:** Multivariable analysis in-hospital mortality

	OR	95% CI	*P*-value
Treatment during CVS phase	0.69	0.3–2.22	0.69
Age	1.04	1.02–1.05	** < 0.001**
BNI score			0.68
1 (reference group)			
2	0.72	0.22–2.29	
3	1.09	0.36–3.27	
4	1.04	0.33–3.29	
5	0.76	0.19–3.14	
HH			** < 0.001**
1 (reference group)			
2	1.5	0.42–5.52	
3	3.39	0.96–11.99	
4	5.35	1.51–18.97	
5	15.12	4.25–53.72	
ICH	1.25	0.78–2.01	0.44
IVH	1.17	0.72–1.89	0.52

Significant *P*-values were marked in bold

*BNI* barrow neurological institute, *HH* Hunt and Hess grade, *ICH* intracerebral haemorrhage, *IVH* intraventricular haemorrhage

### GOS at discharge

Treatment during CVS phase was associated with favourable GOS at discharge in the univariable analysis (OR 0.3, 0.18–0.51 95% CI *p*-value < 0.001). The multivariable analysis was adjusted for age, treatment during CVS phase, BNI score, HH grade, ICH, IVH and smoking. Treatment during CVS phase did not remain significant when adjusting for the above-mentioned variables (OR 0.77, 0.39–1.54 95% CI *p*-value = 0.47). However, age (OR 1.06, 1.04–1.07 95% CI *p*-value < 0.001), HH grade (overall *p*-value < 0.001) as well as ICH (OR 2.69, 1.74–4.18, *p*-value < 0.001), and IVH were significant outcome predictors (OR 1.64, 1.13–2.4 95% CI *p*-value = 0.01; Table 5).

**Table 5:** Multivariable analysis dichotomized GOS at discharge

	OR	95% CI	*P*-value
Treatment during CVS phase	0.77	0.39–1.54	0.47
Age	1.06	1.04–1.07	** < 0.001**
BNI score			0.67
1 (reference group)			
2	0.69	0.26–1.87	
3	0.73	0.28–1.93	
4	0.7	0.25–1.98	
5	1.35	0.37–4.98	
HH			** < 0.001**
1 (reference group)			
2	1.36	0.64–2.89	
3	4.23	1.98–9.05	
4	14.07	6.3–31.43	
5	45.91	18.32–115.05	
ICH	2.69	1.74–4.18	** < 0.001**
IVH	1.64	1.13–2.4	**0.01**
Smoking	0.67	0.37–1.22	0.19

Significant *P*-values were marked in bold

*BNI* barrow neurological Institute, *HH* hunt and hess, *ICH* intracerebral haemorrhage, *IVH* intraventricular haemorrhage

## Discussion

In our study, treatment during the CVS phase was associated with cerebral infarct. However, we did not find an increase of unfavourable outcome according to the dichotomized GOS at discharge or in-hospital mortality among these patients indicating that treatment during CVS phase might be safe.

Treatment during CVS phase was associated with cerebral infarct but not with CVS itself (results not shown). This supports a role of cerebral infarcts separate from CVS as previously described [39]. Other studies also reported on the influence of cerebral infarcts on outcome independently from CVS [40]. The association between treatment during CVS phase and cerebral infarct independently from CVS itself may have several reasons. (1) The increased infarct rate could be treatment related although treatment did not influence the likelihood of cerebral infarct in our cohort and treatment modalities are indeed commonly reported to also influence likelihood for CVS [41]. (2) Another hypothesis is that cerebral infarct in this group of patients could occur due to a delay in intensive care treatment, especially in lower grade aSAH, and not due to the aneurysm occlusion during the vulnerable phase. (3) Treatment delay might enhance the occurrence of microhaemorrhages. Although they might not be extensive enough to be recognized as a rebleeding, they could still increase the risk of cerebral infarcts by increasing blood load. Why this effect is not seen with CVS itself, however, remains unclear. It might be that because patients with CVS can be treated more aggressively after the ruptured aneurysm has been occluded, especially by raising systolic blood pressure, this might lead to a lower CVS but not cerebral infarct rate [28]. In contrast, in patients with untreated aneurysms inadequate CVS therapy may result in an even higher rate of cerebral infarct and poor neurological outcome. (4) Finally, the association might not be as independently as it appears. Patients treated during CVS phase and those not, did have similar CVS rates. Considering the fact that patients treated during CVS phase are usually patients with lower aSAH grades and better clinical state, the finding could be based on a higher rate of CVS in these patients consecutively leading to a higher cerebral infarct rate as can be seen in the multivariable analysis.

Patients treated during CVS phase represented a relatively small number of our overall cohort (10.8%) with even a smaller number actually having CVS when they were treated. However, patients undergoing treatment while having CVS did not influence any of our outcome variables. A previous prospective study reported a higher rate of worse outcome in patients undergoing surgery between days 4 and 10 after the initial event [22]. However, since publication of this study, intensive care treatment has improved and other, e.g., endovascular, treatment options have been developed with a more recent publication indicating no difference in outcome in patients treated between days 4 and 10 [42]. This reflects the modern concept of early or ultra-early occlusion of ruptured aneurysms, so that treatment during the CVS phase is a rarity primarily caused by unintentional delay in confirming the diagnosis in oligosymptomatic and, therefore, lower grade aSAH patients leading to later aneurysm treatment as would be the normal standard of care. However, despite the significantly increased likelihood of cerebral infarct in lower grade aSAH patients, this did not translate into a significant difference regarding functional outcome as measured by dichotomized GOS at discharge in our cohort. Finally, the presented findings could be due to the delayed presentation itself rather than delayed treatment.

In a previously reported meta-analysis, CVS was more frequently observed in patients undergoing clipping compared to coiling [41]. Its influence on functional outcome is less clear. By increasing the rate of CVS, clipping might increase the likelihood for complications as well as unfavourable outcome. However, clipping did not influence any of the outcome parameters in our cohort: when added to the final models (Tables 2, 3, 4, 5) *p*-value was above 0.21 in all outcome analysis (data not shown). In our cohort patients undergoing clipping, even during CVS phase, did not suffer from a worse functional outcome compared to patients undergoing coiling. Similar results have previously been observed [43]. Treatment of aneurysms has strongly shifted from clipping towards endovascular treatment with the majority of unruptured as well as ruptured aneurysms currently being treated by endovascular options. This bears the advantage that especially if treated by endovasculare means during the CVS phase, patients could be treated for potentially present vasospasms at the same time. More than half of the patients treated during the CVS phase exhibited CVS although this was not significantly different from patients treated outside the CVS phase. Of those, 12% underwent vasospasmolysis. In summary, treatment should be adapted to the patient’s specific needs. One of the most important findings in our cohort was that patients treated during the CVS phase undergoing clipping did not have a higher morbidity or mortality rate indicating that there does not appear to be a higher procedural risk.

### Limitations

Our study has limitations: this was a retrospectively conducted analysis meaning that it might exhibit all shortcomings which are associated with retrospective studies. As we excluded patients whose aneurysms were not treated in our department, we might have introduced selection bias. We conducted a sensitivity analysis comparing patient characteristics of those treated in our institutions with those who were not and did not find any significant differences (data not shown). Another limitation is the relatively limited number of patients treated during the CVS period. This might lead to insufficient power to detect an effect although we do not believe that this is the case in our study. We present a large cohort and believe that it is representative of patients suffering from an aSAH. Several studies have suggested delayed cerebral ischaemia (DCI) to be a robust and better outcome variable compared to CVS [35]. Due to the retrospective nature of our study, we specifically decided against the use of DCI as an outcome variable. We decided to use cerebral infarct as it can be objectively analysed using imaging data and does not rely on reporting and documentation. In future studies we suggest to routinely collect the variable DCI in addition to CVS and cerebral infarct to be able to analyse its influence as an outcome as well as independent variable in patients with aSAH. Another point for consideration is the fact that cerebral infarcts occurring within 24–48 h of an intervention in the area of the intervention are not considered to be cerebral infarcts due to DCI and are therefore excluded. This might lead to  underestimation of the influence of cerebral infarction on functional outcome in general. These infarcts might still influence functional outcome significantly. Although consensus is to exclude them from cerebral infarct due to DCI, it might be worth to assess their influence on functional outcome in the future. As we only had limited data on follow-up, functional outcome on follow-up remains to be evaluated.

## Conclusions

Patients with ruptured intracranial aneurysms, including lower grade aSAH, can be safely treated during the CVS phase without increasing the likelihood for complications. In general, aneurysm treatment during CVS phase appears to be relatively safe and should not be postponed due to the subsequent risk of rebleeding and consecutive devastating deterioration.

## Supplementary Information

Below is the link to the electronic supplementary material.

Supplementary file1 (DOCX 33 KB)

## Data Availability

Anonymized data will be shared on request from any qualified investigator, subject to approval of the participating collaborators.

## References

[1] Steiner T, Juvela S, Unterberg A, Jung C, Forsting M, Rinkel G, European Stroke O (2013). European stroke organization guidelines for the management of intracranial aneurysms and subarachnoid haemorrhage. Cerebrovasc Dis.

[2] Al-Khindi T, Macdonald RL, Schweizer TA (2010). Cognitive and functional outcome after aneurysmal subarachnoid hemorrhage. Stroke; J Cereb Circ.

[3] Suarez JI, Tarr RW, Selman WR (2006). Aneurysmal subarachnoid hemorrhage. N Engl J Med.

[4] Nieuwkamp DJ, Setz LE, Algra A, Linn FH, de Rooij NK, Rinkel GJ (2009). Changes in case fatality of aneurysmal subarachnoid haemorrhage over time, according to age, sex, and region: a meta-analysis. Lancet Neurol.

[5] Schievink WI (1997). Intracranial aneurysms. N Engl J Med.

[6] Eriksen N, Rostrup E, Fabricius M, Scheel M, Major S, Winkler MKL, Bohner G, Santos E, Sakowitz OW, Kola V, Reiffurth C, Hartings JA, Vajkoczy P, Woitzik J, Martus P, Lauritzen M, Pakkenberg B, Dreier JP (2019). Early focal brain injury after subarachnoid hemorrhage correlates with spreading depolarizations. Neurology.

[7] Claassen J, Carhuapoma JR, Kreiter KT, Du EY, Connolly ES, Mayer SA (2002). Global cerebral edema after subarachnoid hemorrhage: frequency, predictors, and impact on outcome. Stroke; J Cereb Circ.

[8] Connolly ES, Rabinstein AA, Carhuapoma JR, Derdeyn CP, Dion J, Higashida RT, Hoh BL, Kirkness CJ, Naidech AM, Ogilvy CS, Patel AB, Thompson BG, Vespa P (2012). Guidelines for the management of aneurysmal subarachnoid hemorrhage: a guideline for healthcare professionals from the American heart association/American stroke association. Stroke; J Cereb Circ.

[9] Dreier JP, Lemale CL, Kola V, Friedman A, Schoknecht K (2018). Spreading depolarization is not an epiphenomenon but the principal mechanism of the cytotoxic edema in various gray matter structures of the brain during stroke. Neuropharmacology.

[10] Roos YB, de Haan RJ, Beenen LF, Groen RJ, Albrecht KW, Vermeulen M (2000). Complications and outcome in patients with aneurysmal subarachnoid haemorrhage: a prospective hospital based cohort study in the Netherlands. J Neurol Neurosurg Psychiatry.

[11] Broderick JP, Brott TG, Duldner JE, Tomsick T, Leach A (1994). Initial and recurrent bleeding are the major causes of death following subarachnoid hemorrhage. Stroke; J Cereb Circ.

[12] Hutchinson PJ, Seeley HM, Kirkpatrick PJ (1998). Factors implicated in deaths from subarachnoid haemorrhage: are they avoidable?. Br J Neurosurg.

[13] van Donkelaar CE, Bakker NA, Veeger NJ, Uyttenboogaart M, Metzemaekers JD, Luijckx GJ, Groen RJ, van Dijk JM (2015). Predictive factors for rebleeding after aneurysmal subarachnoid hemorrhage: rebleeding aneurysmal subarachnoid hemorrhage study. Stroke; J Cereb Circ.

[14] Kassell NF, Torner JC (1984). The international cooperative study on timing of aneurysm surgery–an update. Stroke; J Cereb Circ.

[15] Ameen AA, Illingworth R (1981). Anti-fibrinolytic treatment in the pre-operative management of subarachnoid haemorrhage caused by ruptured intracranial aneurysm. J Neurol Neurosurg Psychiatry.

[16] Phillips TJ, Dowling RJ, Yan B, Laidlaw JD, Mitchell PJ (2011). Does treatment of ruptured intracranial aneurysms within 24 hours improve clinical outcome?. Stroke; J Cereb Circ.

[17] Wong GK, Boet R, Ng SC, Chan M, Gin T, Zee B, Poon WS (2012). Ultra-early (within 24 hours) aneurysm treatment after subarachnoid hemorrhage. World Neurosurg.

[18] Kowalski RG, Claassen J, Kreiter KT, Bates JE, Ostapkovich ND, Connolly ES, Mayer SA (2004). Initial misdiagnosis and outcome after subarachnoid hemorrhage. JAMA.

[19] Ois A, Vivas E, Figueras-Aguirre G, Guimaraens L, Cuadrado-Godia E, Avellaneda C, Bertran-Recasens B, Rodriguez-Campello A, Gracia MP, Villalba G, Saldana J, Capellades J, Fernandez-Candil JL, Roquer J (2019). Misdiagnosis worsens prognosis in subarachnoid hemorrhage with good hunt and hess score. Stroke; J Cereb Circu.

[20] Doukas A, Barth H, Petridis KA, Mehdorn M, von der Brelie C (2019). Misdiagnosis of acute subarachnoid hemorrhage in the era of multimodal diagnostic options. Am J Emerg Med.

[21] Cho YD, Han MH, Ahn JH, Jung SC, Kim CH, Kang HS, Kim JE, Lim JW (2015). Simultaneous endovascular treatment of ruptured cerebral aneurysms and vasospasm. Korean J Radiol.

[22] Kassell NF, Torner JC, Jane JA, Haley EC, Adams HP (1990). The international cooperative study on the timing of aneurysm surgery part 2: surgical results. J Neurosurg.

[23] Kassell NF, Torner JC, Haley EC, Jane JA, Adams HP, Kongable GL (1990). The international cooperative study on the timing of aneurysm surgery part 1: overall management results. J Neurosurg.

[24] Bracard S, Schmitt E (2008). Vasospasm and delayed consequences. Interv Neuroradiol.

[25] Dorhout Mees SM, Molyneux AJ, Kerr RS, Algra A, Rinkel GJ (2012). Timing of aneurysm treatment after subarachnoid hemorrhage: relationship with delayed cerebral ischemia and poor outcome. Stroke; J Cereb Circu.

[26] Baltsavias GS, Byrne JV, Halsey J, Coley SC, Sohn MJ, Molyneux AJ (2000). Effects of timing of coil embolization after aneurysmal subarachnoid hemorrhage on procedural morbidity and outcomes. Neurosurgery.

[27] Chyatte D, Fode NC, Sundt TM (1988). Early versus late intracranial aneurysm surgery in subarachnoid hemorrhage. J Neurosurg.

[28] Matias-Guiu JA, Serna-Candel C (2013). Early endovascular treatment of subarachnoid hemorrhage. Interv Neurol.

[29] Jennett B, Bond M (1975). Assessment of outcome after severe brain damage. Lancet.

[30] Kassell NF, Sasaki T, Colohan AR, Nazar G (1985). Cerebral vasospasm following aneurysmal subarachnoid hemorrhage. Stroke; J Cereb Circu.

[31] Pluta RM, Hansen-Schwartz J, Dreier J, Vajkoczy P, Macdonald RL, Nishizawa S, Kasuya H, Wellman G, Keller E, Zauner A, Dorsch N, Clark J, Ono S, Kiris T, Leroux P, Zhang JH (2009). Cerebral vasospasm following subarachnoid hemorrhage: time for a new world of thought. Neurol Res.

[32] Izzy S, Muehlschlegel S (2014). Cerebral vasospasm after aneurysmal subarachnoid hemorrhage and traumatic brain injury. Curr Treat Options Neurol.

[33] Vergouwen MD (2011). Participants in the international multi-disciplinary consensus conference on the critical care management of subarachnoid H vasospasm versus delayed cerebral ischemia as an outcome event in clinical trials and observational studies. Neurocrit Care.

[34] Nassar HGE, Ghali AA, Bahnasy WS, Elawady MM (2019). Vasospasm following aneurysmal subarachnoid hemorrhage: prediction, detection, and intervention. Egypt J Neurol Psychiatr Neurosurg.

[35] Frontera JA, Fernandez A, Schmidt JM, Claassen J, Wartenberg KE, Badjatia N, Connolly ES, Mayer SA (2009). Defining vasospasm after subarachnoid hemorrhage: what is the most clinically relevant definition?. Stroke; J Cereb Circu.

[36] Vergouwen MD, Vermeulen M, van Gijn J, Rinkel GJ, Wijdicks EF, Muizelaar JP, Mendelow AD, Juvela S, Yonas H, Terbrugge KG, Macdonald RL, Diringer MN, Broderick JP, Dreier JP, Roos YB (2010). Definition of delayed cerebral ischemia after aneurysmal subarachnoid hemorrhage as an outcome event in clinical trials and observational studies: proposal of a multidisciplinary research group. Stroke; J Cereb Circu.

[37] Wilson DA, Nakaji P, Abla AA, Uschold TD, Fusco DJ, Oppenlander ME, Albuquerque FC, McDougall CG, Zabramski JM, Spetzler RF (2012). A simple and quantitative method to predict symptomatic vasospasm after subarachnoid hemorrhage based on computed tomography: beyond the fisher scale. Neurosurgery.

[38] Ra S (2008). Multivariable model building.

[39] Findlay JM, Nisar J, Darsaut T (2016). Cerebral vasospasm: a review. Can J Neurol Sci.

[40] Vergouwen MD, Ilodigwe D, Macdonald RL (2011). Cerebral infarction after subarachnoid hemorrhage contributes to poor outcome by vasospasm-dependent and -independent effects. Stroke; J Cereb Circu.

[41] Li H, Pan R, Wang H, Rong X, Yin Z, Milgrom DP, Shi X, Tang Y, Peng Y (2013). Clipping versus coiling for ruptured intracranial aneurysms: a systematic review and meta-analysis. Stroke; J Cereb Circu.

[42] Lawson MF, Chi YY, Velat GJ, Mocco JD, Hoh BL (2010). Timing of aneurysm surgery: the international cooperative study revisited in the era of endovascular coiling. J Neurointerventional Surg.

[43] Hoh BL, Topcuoglu MA, Singhal AB, Pryor JC, Rabinov JD, Rordorf GA, Carter BS, Ogilvy CS (2004). Effect of clipping, craniotomy, or intravascular coiling on cerebral vasospasm and patient outcome after aneurysmal subarachnoid hemorrhage. Neurosurgery.

